# 

**Published:** 1897-08-14

**Authors:** 


					?i Jul uvnniAL/. mrsotu rain?FUKiL, tnereiore BiLbL. mmw^ritarrw:
CADBURY'S ^ CADBURY'S
OOCOA ^1* |p? |fe~ COCOA
" fttoinTble/r,-Z^/.eSt IUrity ^ f>r#1#nt NO ALKALIES USED.
No. 56MYol. XXII. [SSJ.1!] Saturday,
August 14th, 1897
[Subscription Terms,] pK)ICE TWOPENCE.

				

